# Checkpoint inhibitor immune-related adverse events: A focused review on autoantibodies and B cells as biomarkers, advancements and future possibilities

**DOI:** 10.3389/fimmu.2022.991433

**Published:** 2023-01-11

**Authors:** John Taylor, Aesha Gandhi, Elin Gray, Pauline Zaenker

**Affiliations:** ^1^ Centre for Precision Health, Edith Cowan University, Joondalup, WA, Australia; ^2^ School of Medical and Health Sciences, Edith Cowan University, Joondalup, WA, Australia; ^3^ Sir Charles Gairdner Hospital, Department of Medical Oncology, Nedlands, WA, Australia

**Keywords:** autoantibodies, B cells, immune related adverse events, blood-based biomarkers, immune checkpoint toxicities, risk prediction

## Abstract

The use of immune checkpoint inhibitors (ICIs) has evolved rapidly with unprecedented treatment benefits being obtained for cancer patients, including improved patient survival. However, over half of the patients experience immune related adverse events (irAEs) or toxicities, which can be fatal, affect the quality of life of patients and potentially cause treatment interruption or cessation. Complications from these toxicities can also cause long term irreversible organ damage and other chronic health conditions. Toxicities can occur in various organ systems, with common observations in the skin, rheumatologic, gastrointestinal, hepatic, endocrine system and the lungs. These are not only challenging to manage but also difficult to detect during the early stages of treatment. Currently, no biomarker exists to predict which patients are likely to develop toxicities from ICI therapy and efforts to identify robust biomarkers are ongoing. B cells and antibodies against autologous antigens (autoantibodies) have shown promise and are emerging as markers to predict the development of irAEs in cancer patients. In this review, we discuss the interplay between ICIs and toxicities in cancer patients, insights into the underlying mechanisms of irAEs, and the involvement of the humoral immune response, particularly by B cells and autoantibodies in irAE development. We also provide an appraisal of the progress, key empirical results and advances in B cell and autoantibody research as biomarkers for predicting irAEs. We conclude the review by outlining the challenges and steps required for their potential clinical application in the future.

## Introduction

An understanding of the tumor immune response and the role of immune checkpoints in immune tolerance paved the way for the development of a new generation of treatments for cancer patients. The development of immune checkpoint inhibitors (ICIs) has in particular revolutionized the melanoma treatment landscape, with the attainment of durable treatment response and patient survival benefits (1). ICIs re-activate T cells to recognize and destroy cancer cells by blocking the inhibitory signaling pathways necessary for maintaining immune tolerance (2). However, as a consequence of their mechanism of action, ICIs can also cause highly variable non-specific autoinflammation and other tissue directed autoimmune manifestations known as checkpoint toxicities or immune related adverse events (irAEs) (3).

**Figure 1:**
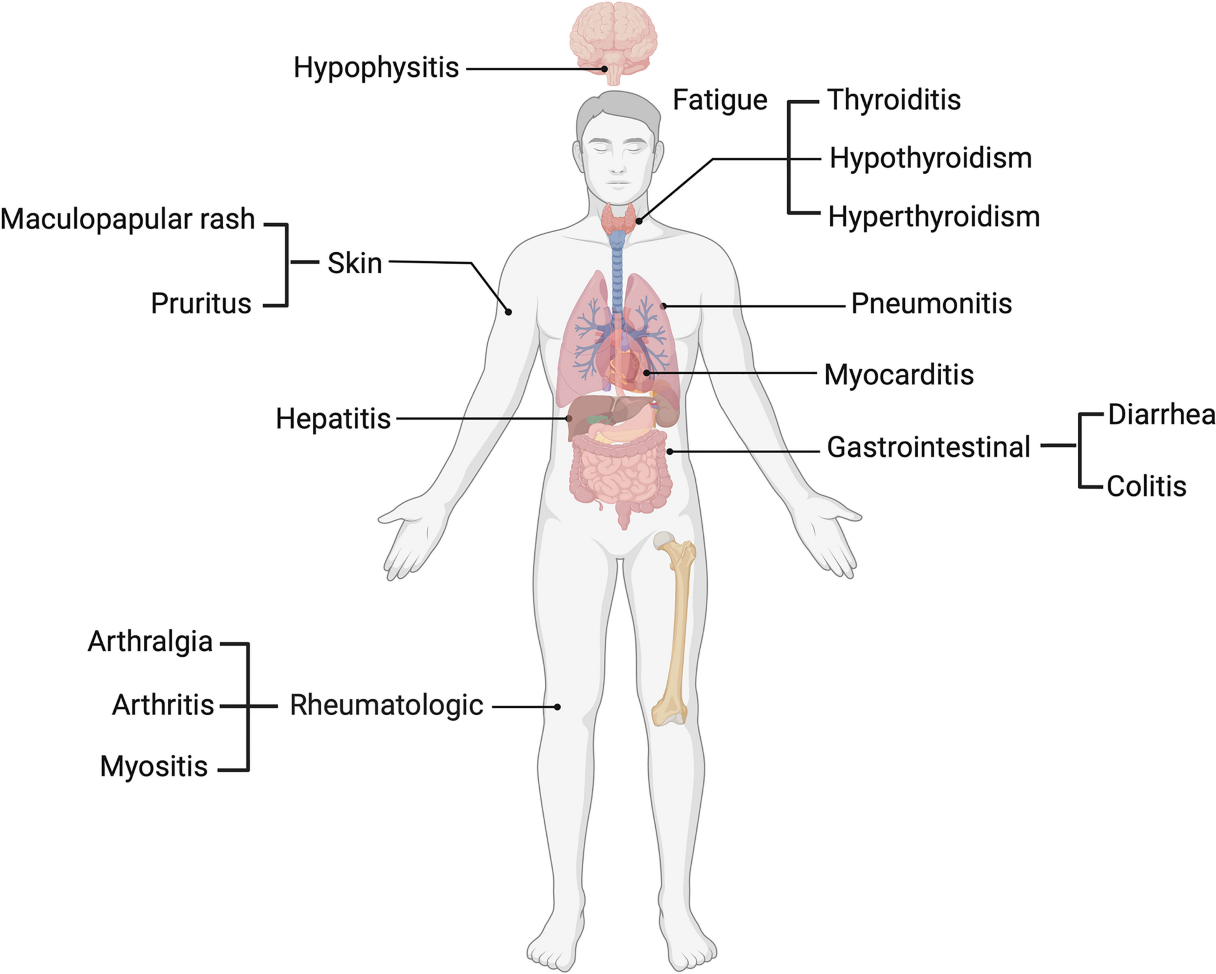
Common immune related adverse events in melanoma patients.

Several ICI clinical trials have outlined a profile of toxicities involving various organ systems, including dermatological, such as maculopapular rash and pruritus (4), pneumonitis in the lungs, gastrointestinal including diarrhea and colitis (5) and hepatic toxicity, which presents with increases in circulatory liver enzymes such as aspartate (AST) and alanine transaminases (ALT) (5). Endocrine toxicities such as thyroiditis and hypophysitis (6), rheumatologic toxicities including arthralgia and arthritis (7) and other non-organ specific events such as fatigue (8) have also been reported (**Figure 1**). IrAEs are graded and managed according to the common terminology criteria for adverse events (CTCAE), and patients may be classified based on mild (grade 1 and 2) or severe (grade 3-5) irAEs. Among melanoma patients, more than 50% who undergo treatment with ICIs experience severe grade (grade 3-5) irAEs (9).

Although irreversible complications can occur, irAEs are generally reversible and can be managed with immunosuppressive drugs and corticosteroids (10). However, this is largely dependent on the early recognition, and prompt treatment of the toxicity. In this context, biomarkers that are predictive of which patients are likely to suffer irAEs prior to or early during treatment have gained importance to support the early irAE detection and its management.

In an effort to identify biomarkers for the prediction of irAEs, numerous biomarkers have been pursued (11). Herein, we focus on the role of the humoral immune response involving B cells and autoantibodies (AAbs). In this review, we discuss ICIs and irAEs in various cancers, the underlying mechanisms of irAEs, and the involvement of AAbs and B cells in irAE development. We also discuss the evidence that supports the validity of AAbs and B cells as biomarkers of irAEs and address the challenges encountered for their clinical utilization and new avenues for research.

We relied on original published articles and limited our search in NCBI PubMed to the following keywords “autoantibodies as biomarkers of checkpoint toxicities”, “immune related adverse events”, “checkpoint toxicities”, “autoantibodies and B cells as biomarkers of immune related adverse events”, “B cells in immune related adverse events’’, “B cell profiling in cancer”, “autoantibody profiling in cancer” and “side effects of immune checkpoint inhibition”.

## Breaking tolerance: A double-edged sword

**Figure 2:**
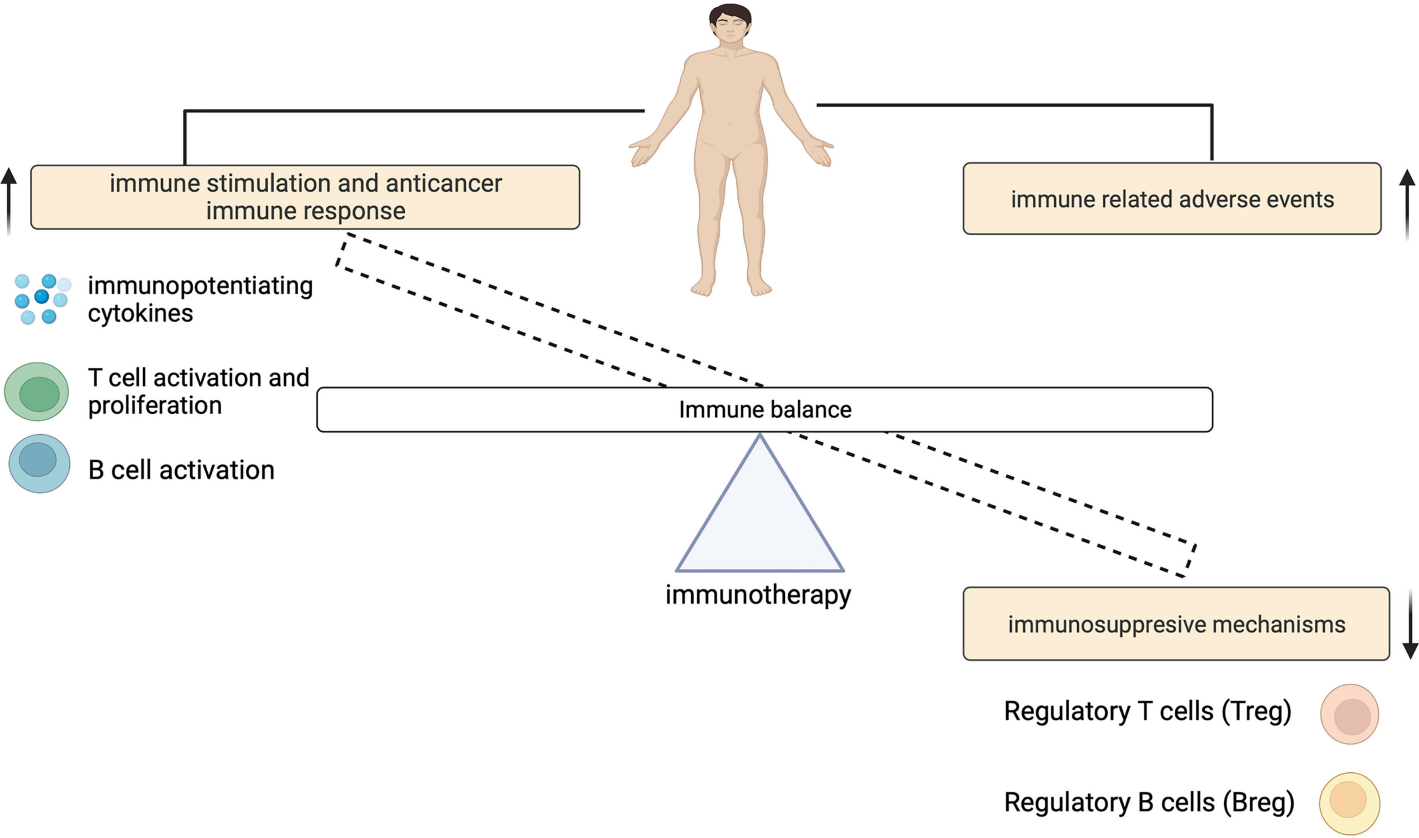
An imbalance of the immune system can cause immune related adverse events.

**Figure 3:**
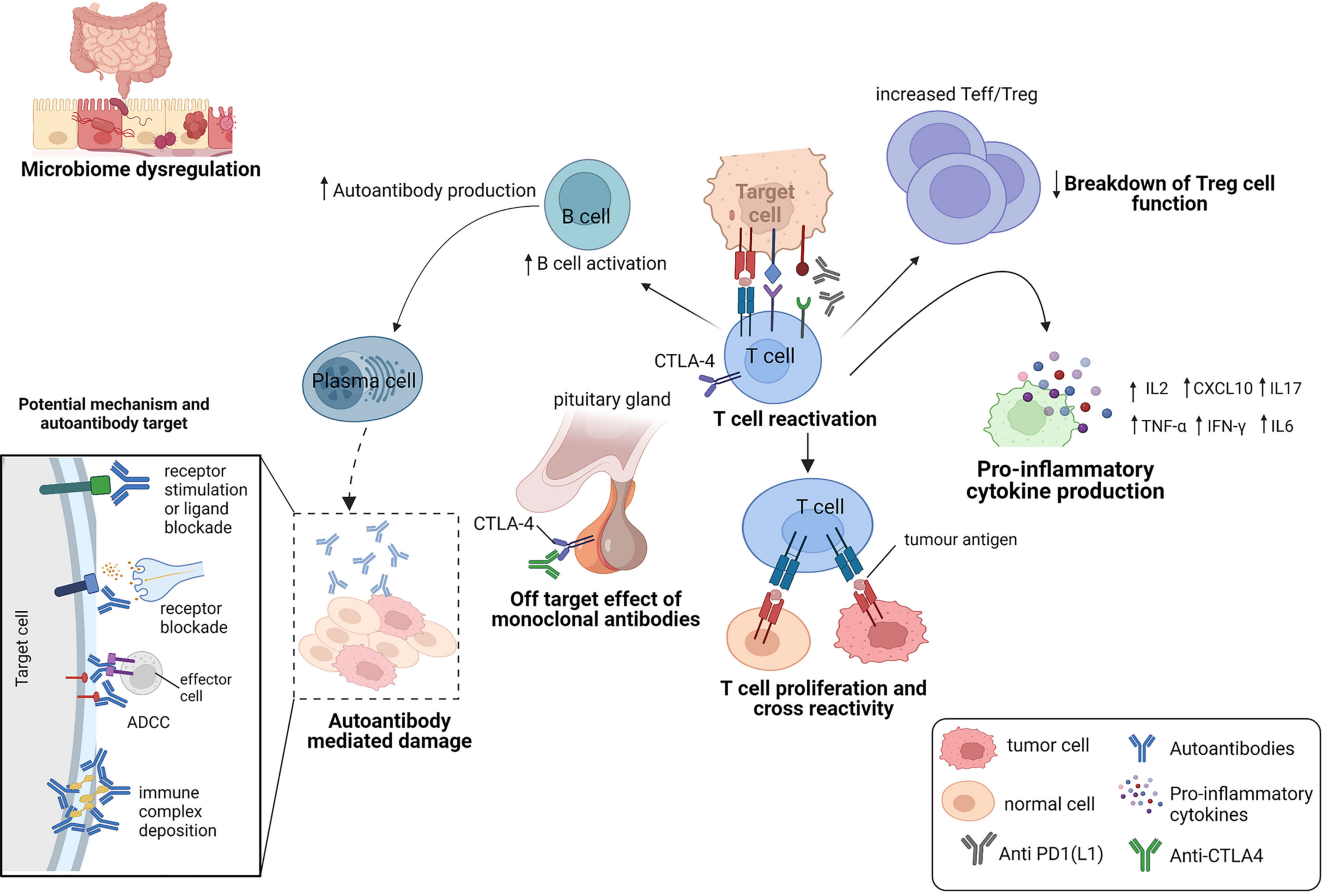
Potential mechanisms of immune related adverse events.

The contribution of ICIs to both controlling tumor growth and causing irAEs, highlights a dynamic interplay that exists between this treatment outcomes (**Figure 2**). The physiologic function of immune checkpoints is to maintain immune tolerance (10). Unlike cytotoxic T-lymphocyte-associated antigen 4 (CTLA-4) which suppresses T cell activation at the initial priming phase of T cell activation (12, 13), engagement of programmed cell death 1 (PD-1) with its ligands (programmed death-ligand 1 (PD-L1) and also PD-L2) inhibits the T cell effector phase. This results in the failure in the proliferation of effector T cells and the production of cytokines such as interleukin-2 (IL-2), tumor necrosis factor-α (TNF-α) and interferon gamma (IFN)-γ (14). During ICI therapy, this inhibitory control mechanism is interrupted which enables the immune recognition and destruction of cancer cells. But because this process is not antigen specific, ICIs may also reactivate the immune system to self-antigens, capable of causing irAEs (**Figure 3**).

## Mechanisms of immune-related adverse events

IrAEs are thought to be primarily mediated by an overactivation of the immune system. However, the immune components that drive them are heterogenous and may be affected by other factors such as the gut microbiome dysregulation, pre-existing autoimmune disease, host genetics and environmental cues (15). Here, we describe the mechanisms that have been implicated in the current literature (**Figure 3**).

### Off-target effect of monoclonal antibodies

The expression of immune checkpoints in normal tissues has been implicated in the development of organ-specific irAEs (16, 17) (**Figure 3**). An example is hypophysitis, which is rarely seen following anti-PD-1 therapy. The modelling of hypophysitis in C57BL/6J mice provided evidence to support the off-target effects of monoclonal antibodies as a potential mechanism for ICI toxicity (16). In this study, CTLA-4 expressed in the hypothalamic and pituitary tissues in mice, was reported to serve as a target for monoclonal antibody (anti-CTLA-4)-mediated injury, complement activation and subsequent organ damage of the pituitary (16). Caturegli et al. (17) also analyzed the pituitary expression of CTLA-4 in 6 cancer patients treated with anti-CTLA-4. They found that CTLA-4 expression was highest in severe hypophysitis patients compared to other pituitary glands. A higher CTLA-4 expression was associated with extensive destruction of the pituitary *via* immune mechanisms including T cell infiltration, antibody-dependent complement fixation and phagocytosis. They showed that higher levels of CTLA-4 in the pituitary can result in hypophysitis through T cell and antibody dependent immune mechanisms, during CTLA-4 blockade (17).

### Immune system reactivation from checkpoint inhibition

The chronic exposure of antigens to infiltrated T cells (TILs) in the tumor microenvironment (TME) can also result in an exhausted T cell phenotype (14), characterized by the expression of multiple inhibitory cell surface checkpoint molecules (such as PD-1), changes in the metabolic and epigenetic profiles of T cells (18) and a loss of T cell effector function (19). This expansion of exhausted T cells (with upregulated expression of immune checkpoints) results in limited damage to cancer cells (19), while preventing any autoimmune damage to the patient (3). CTLA-4 also reduces immune system overactivation, by binding to B7 proteins (CD80 and CD86) present on antigen presenting cells (13). This attenuates the MHC-TCR signaling, restraining T cells from overactivation. During cancer therapy, ICIs counteract these immune checkpoints in the TME to reinvigorate the immune system for cancer cell recognition and attack. The re-activation of the immune system to fight cancer cells by altering this highly important regulatory network, has been shown to present with an unspecific and unpredictable inflammatory feedback that worsens systemic autoimmunity and the occurrence of irAEs.

### Cross reaction of T cells for a common antigen between tumor and healthy cells

The capability of a T cell receptor (TCR) to bind more than one peptide has also been shown to be very important in autoimmune responses (20). Peptides can activate autoreactive T cells due to the structural similarity between seemingly unrelated peptide-loaded major histocompatibility complexes (pMHC) and cause autoimmune diseases (21–23). Several studies suggests that the off target T cell recognition of tumoral antigens on healthy cells can cause adverse events in patients treated with ICIs (24) (**Figure 3**). Studies have reported tumoral T cell infiltration into healthy tissues (25), identical TCR sequences in both tumor and healthy tissue (26) and similar shared antigen recognition in tumor and affected organs in the occurrence of irAEs (27–30). In these studies, epitope spreading was shown to be one of the mechanisms, that drive the off-target T cells (31, 32). Treatment with anti-CTLA-4 was shown to broaden the peripheral TCR repertoire in melanoma patients, that differed for those with irAEs and those without (33). Similar early T cell diversification of the TCR repertoire by CTLA-4 blockade was reported by Oh et al. (34). In these studies, the expansion of the TCR repertoire was shown to correlate with the occurrence of irAEs. Altogether, these studies demonstrate that ICIs can trigger irAEs by re-activating and *de-novo* inducing T cell clones, some of which not only recognize tumor antigens but also self-antigens.

### Downregulation of regulatory T cell functions

IrAEs can also occur as a consequence of a downregulation of regulatory T cell (Treg) function. A subset of Tregs express CTLA-4, and maintain immune tolerance by immune response suppression, either by producing inhibitory cytokines, mediating T cell metabolic disruption, or by modulation of dendritic-cell (DC) maturation or function (35). CTLA-4 blockade was shown to impair Treg cell function and survival (36, 37), which was shown to increase the ratio of effector T cells to Treg cells (Teffs/Tregs) (37). An altered balance between Tregs and T effector cells (Teffs) was also shown to result in a loss of peripheral tolerance (38), that can lead to the development of autoimmune disease in various cancers (39).

A randomized controlled trial in cancer patients, reported decreased numbers of circulating Treg cells in patients who experienced irAEs on anti-CTLA-4 treatment (40). However, another study (41) did not report intratumoral depletion of Treg cells in cancer patients treated with anti-CTLA-4 therapies.

Despite this, it is plausible that targeting of regulatory T cells by ICI therapy can lead to an enhanced T cell effector proliferation and survival capable of killing tumor cells and also cause autoimmune side effects. PD-1 and PD-L1 also play a role in the development and survival of Treg cells (42, 43) and their inhibition has been shown to result in decreased Treg cell function that can cause irAEs.

### Proinflammatory cytokine production from T cell activation

A range of studies suggest that the release of cytokines during ICI therapy may also play a role in the development of irAEs. Cytokines regulate the immune system activity (44, 45) and provide an enabling environment for the function of other immune cells. They mediate several cell signaling pathways for T cell activation, cytotoxicity and survival (46, 47) and B cell differentiation into antibody producing plasma cells (48, 49). However, proinflammatory cytokines can trigger a systemic inflammatory response, which can lead to the occurrence of irAEs. For example, CTLA-4 blockade to promote T cell activation was shown to release pro-inflammatory cytokines in circulation (50). Melanoma patients who were treated with anti-CTLA-4, showed increased levels in the IL-17 producing CD4+ Th17 cells following treatment (51). IL-17 has been shown to mediate irAEs (2) and has been implicated in several autoimmune diseases (52). In one study, increased baseline circulation of IL-17 was found to correlate with the occurrence of severe colitis among advanced melanoma patients receiving neoadjuvant ipilimumab (50). Lim and colleagues (53) also reported elevated circulatory levels of pro-inflammatory cytokines in metastatic melanoma patients who experienced irAEs on combination therapy. Patients who developed irAEs also showed lower serum levels of C-X-C motif chemokine ligand 9 (CXCL9), CXCL10, CXCL11 and CXCL19 at baseline and higher levels of CXCL9 and CXCL10 post treatment in patients who did not develop any irAEs (54). Several studies have demonstrated IL-6 association with several irAEs (55–57). IL-6 is associated with the polarization of CD4^+^T cells to Th17 cells (46). IL-6 is also associated with an increase in the acute phase proteins such as C-reactive protein (CRP), which is also a marker of inflammation. Reduction in serum IL-6 levels was correlated with resolution of ICI-induced colitis, in response to corticosteroids and reduction of infiltrating Th17 cells in the inflamed sections of the colon (58).

Cytokine inhibitors such as infliximab (anti-TNF-a), tocilizumab (anti-IL-6R) and secukinumab (anti-IL-17a) that are administered secondary to corticosteroids in severe irAEs, have also demonstrated efficacy in resolving irAEs (59, 60). Tocilizumab binds to IL-6 receptors, and inhibits the IL-6 receptor complex signaling that can cause systemic and local inflammation (46) and also B cell differentiation (49). Recently, inhibition of IL-17A was shown to significantly reduce the development of thyroid irAE in ICI-treated tumor-bearing mice (61). Altogether, these findings suggest an important role of cytokines in the occurrence of irAEs.

### Microbiome dysregulation

The contribution of the gut microbiome to autoimmune diseases has gained momentum in recent years and the gut microbiome appear to also regulate toxicities that arise from the use of ICIs (62, 63) In addition to their key roles in metabolism and absorption, gut microorganisms are involved in the maintenance of the host immune homeostasis (such as by bacteroidetes which can stimulate the differentiation of Treg cells) (63). Dysregulation of the composition of the gut microbiome has been implicated in the occurrence of irAEs and two published studies provided evidence to support this suggestion. Chaput et al. (57), investigated the baseline composition of the gut microbiome of metastatic melanoma patients on ipilimumab, and identified two microbial populations which were associated with colitis. They identified a lower population of *Bacteroidetes* and a higher abundance of *Firmicutes* which was significantly associated with the occurrence of colitis. Dubin et al. (64) also reported an increase in fecal *Bacteroidetes* in baseline samples of metastatic melanoma patients who did not develop any colitis on ipilimumab treatment.

A study by Andrews et al. (62) also found a higher abundance of *Bacteroides intestinalis* in metastatic melanoma patients who developed severe grade irAEs on combination therapy. In this study, an upregulation of mucosal IL-1β (which is a key mediator of autoinflammatory responses) was reported in patient samples of colitis and in pre-clinical models. The successful treatment of ICI-induced colitis with fecal transplantation (a method that enables the modulation of the microbial composition in the gut) also lends support to the role of the gut microbiota in the occurrence of irAEs (65). Despite the possible association between the dysregulation of the gut microbiome and the development of irAEs, further studies are required to determine their underlying mechanisms.

### Autoantibody action

Breakdown of self-tolerance mechanisms against autoantigens results in the initiation of antibody effects (66, 67) and it is becoming clear that AAbs can mediate irAEs by causing systemic inflammation that results in cellular and tissue injury (67) (**Figure 3**). AAbs may also cause autoimmune phenotypes by inhibiting the function of their target proteins either by stimulating protein receptors or blocking stimulation by its natural ligand (67). In Graves’ disease, for example, the binding of AAbs against the thyroid stimulating hormone receptor on thyroid cells was shown to cause the abnormal production of thyroid hormones which can lead to hyperthyroidism in patients (68). In a study by Osorio et al. (69), antithyroid antibodies were frequently found in patients who developed ICI-induced thyroid dysfunction.

Other possible pathogenic roles include activating the complement system and causing organ damage *via* the formation and deposition of immune complexes (67). In another retrospective study in advanced NSCLC patients treated with anti-PD-1, patients positive for any AAbs were significantly more likely to develop irAEs than those who were AAb‐negative (70).

In metastatic melanoma patients who were treated with ICIs, increased levels of pre-existing AAbs were shown to preceed the development of severe grade irAEs (71). 4 out of 5 cancer patients who had pre-existing antiacetylcholine receptor AAbs, were also shown to develop myositis on anti-PD-L1 treatment (72).

The presence of pre-existing AAbs is characteristic of many autoimmune diseases including rheumatoid arthritis and systemic lupus erythematosus and cancer patients with pre-existing autoimmune disease may also be prone to the development of irAEs or an autoimmune disease flare (73–75). A systematic review of cancer patients with autoimmune disease showed that, 75% of patients who were treated with ICIs had exacerbation of their pre-existing autoimmune disease, development of a *de novo* irAE or both (75). In this study, patients who were receiving treatment for pre-existing autoimmune disease when ICI therapy started had fewer irAEs compared to those who were not receiving any treatment (75). An increase in autoimmune disease flare was also reported by Menzies et al. (76) among patients with active autoimmune disease compared with patients with inactive disease. Altogether, suggesting an underlying predisposition to the occurrence of irAEs.

Success with B cell depleting agents such as rituximab (anti-CD20) (77, 78), also support the concept that B cells and antibodies may play a role in the occurrence of irAEs. Rituximab reduces B cell differentiation, and has been useful in the management of both classical autoimmune diseases (77) and irAEs (78). While focus of most of the published literature has been on assessing IgGs in checkpoint toxicities, a brief case report by Zaenker, Prentice and Ziman (79) reported higher levels of tropomyosin IgA AAbs which was associated with the occurrence of ICI-induced myositis in a uveal melanoma patient. Serum IgA has been implicated in autoimmunity and inflammation, and their aggregation can be detrimental, capable of inducing inflammatory diseases (80, 81). Overall, while the mechanisms underlying the role of AAbs in the development of irAEs remains unclear, their presence has been implicated in irAEs which can suggest some important pathology element. Nevertheless, whether AAbs are causal, or just correlative of an unspecific B cell activation from ICI therapy is still not clear.

## ICI modulation of B cells and autoantibody expansion in irAEs

Studies have showed that ICIs can impact other immune cells (82), which can also explain the extent of adverse events associated with ICIs. Here we describe the expression of CTLA-4 and PD-1/PD-L1 proteins in B cells and the role of ICI in this immune cell populations in the occurrence of irAEs.

Yang et al. (83) demonstrated the expression of CTLA-4 in mouse B-1a cells and showed that deletion of CTLA-4 from B cells results in mice that develop AAbs, T follicular helper (Tfh) cells, germinal centers in the spleen, and autoimmune outcomes. This impaired immune homeostasis was demonstrated to be as a result of B cell dysfunction upon loss of CTLA-4 (83). This aligns with earlier studies implicating CTLA-4 in the control of B cell responses and antibody production by modulating T follicular helper (Tfh) cells (84). A study published in 2014 by Sage et al. (84) showed that, a deletion or dysfunction of CTLA-4 in mice increased Tfh and T follicular regulatory (Tfr) cell numbers with improved B cell responses. The loss of CTLA-4 on the Tfh cells increased B cell responses, whereas loss of CTLA-4 on Tfr cells resulted in increased antigen-specific antibody responses.

Thibult et al. (85), also demonstrated the expression of PD-1 on major human B-cells subsets. They showed that blockade of the PD-1 pathways increased the proliferation and activation of B cells and the production of inflammatory cytokines. Indeed, since its discovery, PD-1 has been shown to play a role in the negative regulation of B cell proliferation and differentiation (86).

In patients with melanoma treated with combination ICI therapy, changes in B cell populations was shown to increase the likelihood of irAEs (87). An overall decrease in circulatory B cells and an increase in plasmablasts and CD21^lo^ PD-1+ B cells was observed (87). Another study also reported an enhanced increase in circulating plasmablasts in cancer patients on anti-CTLA-4 (88) who experienced various autoimmune outcomes. In this study, sequencing of immunoglobulins produced by the plasmablasts showed evidence of somatic hypermutation, clonal expansion and class switching. In humans with germline mutations in CTLA-4 who experienced autoimmune disease, increases in CD21^lo^ B cells were also found in circulation with an overall decrease in total B cells (89).

The recognition of PD-1 and CTLA-4 as regulators of B cell activation provide clues as to how ICIs may affect the balance of B cells and AAb production in the occurrence of irAEs.

## Autoantibodies and B cells as biomarkers of immune related adverse events

Generally, an ideal biomarker of irAEs will need to be highly sensitive, specific, and precise to distinguish between toxicity groups. It should be robust for accurate prediction, allowing for early irAE diagnosis, determining severity and risk prior to therapy. Predicting the onset of toxicities prior to therapy has become an important clinical need to support patient selection and personalized monitoring. However, history of autoimmune disease remains the clinical risk stratification parameter, for ICI toxicities in cancer patients. Although other routine clinical laboratory assays, that assesses blood enzymes and hormones, may be useful, they often reflect end organ-toxicity, and may not provide predictive value. Here, we provide a summary of the extent of research on AAbs and B cells as toxicity biomarkers in cancer.

### Autoantibodies as biomarkers of immune related adverse events

Antibodies have been found that recognize several tumor and self-antigens, including overexpressed or aberrantly expressed antigens, modified proteins and intracellular molecules, and neoantigens (originating from mutations and alternative splicing events) that can drive humoral responses (66). Serum AAb levels against tumor-associated and self-antigens have been proposed as biomarkers for cancer detection (90), prognosis (91), ICI response and toxicities (reviewed here, **Table 1**).

**Table 1:** Studies on autoantibodies as biomarkers of immune related adverse events.

Author, year (reference)	AAb specificity	Sample size (with irAEs)	Tumor type	Time point	Method	ICI type/therapy	Diagnostic accuracy (Sensitivity, Specificity and AUC)	Odds ratio (CI) and significance	Study design	Type of Adverse events	Summary of findings
Maekura et al. (2017) (92)	Anti-Tg Anti-TPO	64 (5)	NSCLC	Baseline and until 4 months of treatment	Electrochemiluminescence immunoassay.	anti-PD-1	–	p value < 0.001 p value = 0.002	Retrospective study	hypothyroidism	TgAb and TPOAb were positive for the occurrence of hypothyroidism
Kimbara et al. (2018) (93)	Anti-Tg	168 (35)	Solid tumors-melanoma and NSCLC	Baseline and on treatment	Biochemistry test (blood analysis of antithyroid antibodies)	anti-PD-1	–	OR_adj_ = 26.5 (95% CI, 8.18-85.8) p value = <0.001	Retrospective study	Thyroid dysfunction	Baseline levels of TgAb was significantly associated with the development of thyroid dysfunction
Gowen et al. (2018) (94)	Various autoantibodies	75 (63)	Metastatic melanoma	Baseline	HuProt Microarray	anti-CTLA-4, anti-PD-1, anti-CTLA-1/PD-1 combination	ACUR: > 90%Spec:, Sensi: ≥ 0.89	–	Prospective study	Various irAEs	Baseline AAbs was predictive of the occurrence of irAEs
Hasan et al. (2019) (95)	Anti-BP180IgG	40 (16)	NSCLC	Baseline	ELISA	anti-PD-1 and Anti- PDL1	–	p value = 0.04	Prospective study	Skin irAEs-rash and pruritus	Elevated baseline levels of anti-BP180 IgGs correlated with the development of skin irAEs
Tahir et al. (2019) (96)	Anti-GNAL Anti-CD74	20 (5) 32 (10)	Solid tumor-prostate cancer, renal cell carcinoma, melanoma, bladder, and pancreatic cancer	Pretreatment and posttreatment	Serological analysis of recombinant CDNA expression libraries	anti-CTLA-4, anti-PD-1, anti-CTLA-1/PD-1 combination	AUC = 0.79 AUC= 0.76	OR = 2.66 (95% CI 1,14-7.29) p value = 0.02 OR = 1.25 (95% CI 1.03-1.52) p value = 0.03	Nested prospective study	Hypophysitis Pneumonitis	Pre-treament anti-GNAL and anti-CD74 predicted and was associated with hypophysitis and pneumonitis respectively
Toi et al. (2019) (70)	rheumatoid factor, antinuclear antibody, antithyroglobulin, and antithyroid peroxidase	137 (66)	NSCLC	Baseline	Various immunoassays	anti-PD-1	–	OR =0.25 (95% CI, 1.59-6.65) p value = 0.001	Retrospective study	Various irAEs	Baseline pre-existing antibodies was associated with the occurrence of irAEs
Yoneshima et al. (2019) (97)	Antinuclear antibodies (ANA)	83 (27)	NSCLC	Baseline	Indirect immunofluorescence assay	anti-PD-1	–	p value > 0.05	Retrospective study	Various irAEs	Occurrenceof irAEs did not differ significantly among patients positive and negative for pre-existing ANAs
Kurimoto et al. (2020) (98)	Anti-Tg	26 (13)	melanoma, gastric cancer, renal cell carcinoma, urothelial cancer, and NSCLC	Baseline and 4 weeks after first dose of ICI therapy	Electro chemiluminescent immunoassay	anti-CTLA-4, anti-PD-1, anti-CTLA-1/PD-1 combination	–	p value ≤ 0.05	Prospective study	Thyroid dysfunction	Early increase in TgAb (≤ 4 weeks) was associated with the development of thyroid dysfunction
Sakakida et al. (2020) (99)	Antinuclear antibodies (ANA)	191 (73)	Solid organ cancers-NSCLC, melanoma, renal cell carcinoma, head & neck cancer, gastric cancer, urothelial cancer	Baseline and follow up	Indirect immunofluorescence assay	anti-PD-1, anti-PD-L1	–	p value > 0.05	Retrospective study	Various irAEs	Pre-existing ANAs was not associated with the development of irAEs
Iñigo Les et al. (2021) (100)	Antinuclear (ANA), anti-neutrophil cytoplasmic (ANCA), anti-thyroid antibodies (ATA) and rheumatoid factor (RF)	26 (16)	Solid organ cancers-NSCLC, melanoma, renal cell carcinoma, head & neck cancer	After first dose of ICI therapy	Various immunoassays	anti-PD-1	ACUR: 80.8%Spec: 70% Sensi: 87.5%	OR =46.61 (95% CI, 2.48- 876.10) p value = 0.01	Retrospective study	Various irAEs	AAbs was predictive of irAEs
Ghosh et al. (2022) (101)	Antinuclear (ANA), rheumatoid factor (RF), and anti-cyclic citrullinated peptide antibody (anti-CCP).	60 (55)	Metastatic melanoma	Baseline and 6 weeks after ICI initiation	Multiplex assay	anti-PD-1/CTLA-4 combination	–	p value = 0.13	Retrospective study	Various irAEs	Baseline levels of AAbs was not associated with irAEs
Zhang et al. (2022) (102)	Antinuclear antibodies (ANA)	159 (46)	Metastatic NSCLC	Baseline and every 2 months follow-up	Indirect immunofluorescence assay	anti-CTLA-4, anti-PD-1, anti-PD-L1	–	OR =4.9 (95% CI, 1.45- 16.52) p value = 0.01	Retrospective study	Various irAEs	Pre-existing ANAs was associated with the occurrence of irAEs
Tang et al. (2022) (103)	Antinuclear antibodies (ANA)	177 (82)	NSCLC, gastric cancer, head and neck cancer, eosophageal cell squamous carcinoma	Baseline	Indirect immunofluorescence assay	anti-PD-1, anti-PD-L1	–	p value > 0.05	Retrospective study	Various irAEs	Pre-existing ANAs was not associated with the occurrence of irAEs
Barth et al. (2022) (104)	Various rheumatologic antibodies	44 (5)	Solid organ cancers-NSCLC, renal cell carcinoma, head & neck cancer, gastric cancer, urothelial cancer, colorectal cancer	Baseline and 8-12 weeks of treatment	Various immunoassays	anti-PD-1, anti-PD-L1, anti-PD-1/CTLA-4 combination	–	OR = 0.702 (95% CI, 0.105–4.674), p = 0.714	Prospective study	Various irAEs	Patients with positive AAb titres showed no increased risk for the development of irAEs

CI, confidence interval; OR, odds ratio; OR_adj_, adjusted odds ratio; -, not reported in the study; AAbs, autoantibodies; ACUR, accuracy; Spec, specificity; Sensi, sensitivity; AUC, area under the curve; Abs, antibodies.

For example, in a group of 5 NSCLC patients undergoing treatment with nivolumab, Maekura et al. (92) identified baseline levels of anti-thyroid peroxidase (TPO) and anti-thyroglobulin (Tg) that were positive for the occurrence of hypothyroidism. Similar findings were recorded by Kimbara et al. (93) in melanoma and NSCLC patients, and Kurimoto et al. (98) in various cancer patients on anti-CTLA-4, anti-PD-1 and anti-CTLA-1/PD-1 combination therapy. Another study conducted by Toi et al. (70) also identified pre-existing rheumatological antibodies particularly, rheumatoid factor (RF), antinuclear antibodies (ANA), anti-Tg and anti-TPO, that were independently associated with various irAEs in a multivariate analysis. In this study, skin and thyroid disorders were frequently found in patients with pre-existing RF and antithyroid antibodies respectively. Zhang et al. (102) also found an association between pre-existing ANAs (at a titer ≥1:320) and various irAEs, and an increase in adverse skin reactions among ANA-positive patients compared to negative patients.

Campochiaro et al. (105), examined a panel of rheumatological AAbs prior to glucocorticoid use and at follow-up in a group of cancer patients, who presented with rheumatic irAEs following ICI therapy. In this study, AAb positivity was the only factor that was associated with the need for an add-on therapy (immunosuppressants) during follow-up. By considering the requirement of an add-on therapy as a surrogate for disease severity, the findings of this study suggested the utility of rheumatological AAbs to predict disease severity or a high disease activity, even though none of the patients were tested for AAbs prior to the start of ICI therapy.

At the time of this review, only one retrospective study in a small group of cancer patients had demonstrated the diagnostic performance (accuracy, sensitivity and specificity) of a classical rheumatological AAb battery to predict toxicities (100). Despite the study limitation of small sample size, classical AAbs composed of ANA, antineutrophil cytoplasmic antibody (ANCA), RF and antithyroid antibodies (ATA) measured after first dose of anti-PD-1(nivolumab), showed a diagnostic accuracy of 80.8% with a sensitivity and specificity of 87.5 and 70% for toxicity prediction (100).

While these findings may be encouraging, conflicting results exist in the utility of prototypical rheumatological AAbs as predictors of irAEs. Ghosh et al. (101) examined the expression of several rheumatological AAbs (ANA, RF and anti-CCP (cyclic citrullinated peptide)) among advanced melanoma patients on combination (ipilimumab/nivolumab) therapy using a customized microarray at baseline and 6 weeks during therapy. Although AAb levels increased after 6 weeks of treatment in patients with irAEs, baseline levels of ANA, RF and anti-CCP, were not predictive of specific irAEs. ANA, RF and anti-CCP did not show any significant difference (p = 0.13) between serological negative and positive patients for irAE development, onset, severity or survival (101). Similarly, although De Moel et al. (106) also recorded an increase in rheumatologic AAbs from baseline in late-stage melanoma patients (who were antibody negative pre-treatment), no association with any irAEs was observed. Several reports have also found no significant association between pre-existing ANAs in various cancer patients prior to ICI therapy, with the occurrence of irAEs (97, 99, 103, 104). Despite the temporal resemblance of classic autoimmune diseases with irAEs, it appears that their mechanisms might be different and whether an increase or decrease in classic rheumatological AAbs is beneficial for the prediction of irAEs remains controversial and requires further investigation.

Several other studies involving various analytical approaches have identified antibody targets that correlate with irAEs. Duarte et al. (71) identified an increase in a repertoire of AAb targets in a cohort of melanoma patients on ipilimumab, that correlated with the occurrence of irAEs. Interestingly, increase in serum AAbs from baseline, preceded the development of irAEs. Using a high throughput protein microarray, Gowen et al. (94) also identified differential pre-treatment AAb proteomic profiles that predicted the occurrence of irAEs among late-stage melanoma patients with an accuracy of >90%. Findings from this study provided further evidence to suggest the existence of a subclinical autoimmune profile in a subset of ICI-treated patients, that renders them susceptible to the development of irAEs. Other case observations and organ related AAb targets have been identified that correlate with irAEs. Tahir et al. (96) screened baseline plasma samples from various cancer patients who developed ICI-induced hypophysitis and pneumonitis against a cDNA expression library of the brain and lungs. The authors identified increased pre-treatment anti-guanine nucleotide-binding protein G subunit alpha (anti-GNAL) which was associated with hypophysitis (OR = 2.66; 95% CI 1,14-7.29 p = 0.02, AUC = 0.79), and anti-CD74 levels with pneumonitis (OR = 1.25; 95% CI 1.03-1.52 p = 0.03, AUC = 0.76). GNAL has been shown to play a significant role in the activation of the cAMP signaling pathway; a pathway important in cell proliferation, hormone synthesis and secretion in the pituitary (107).

As observed in other studies (98, 101, 106), an increase in fold change expression of both GNAL and CD74 from pre-treatment to post treatment was recorded, which discriminated toxicity groups (healthy vs irAEs) with an AUC = 1 for both markers. In addition, post treatment levels of both markers (i.e anti-GNAL and anti-CD74) discriminated toxicity groups with an AUC of 0.92 and 0.95 respectively. Hassan et al. (95) also demonstrated the co-expression of the B cell targeted antigen bullous pemphigoid 180 (BP180) between healthy skin and NSCLC tissues and identified elevated anti-BP180 IgG at baseline that correlated with skin toxicities (p = 0.04), therapy response (p = 0.01) and overall survival (p = 0.04). While the exact roles of toxicity-associated antibodies remain elusive, a subset of AAbs may be involved in irAEs, and their measurements could be useful for the prediction of ICI toxicities.

### B cells as biomarkers of immune related adverse events

Recent work on the role of B cells have led to the recognition of their importance in the immune surveillance process and as cancer biomarkers (108–110). While research is ongoing on the exact roles of B cells in the tumor environment, several studies have demonstrated their potential as biomarkers of response and survival in various cancers (111–114). Based on various user-defined immune phenotypes (markers), various B cell subsets including activated B cells, germinal center B cells, plasmablasts, plasma cells, transitional and memory B cells have been found in cancer patients treated with ICIs with various predictive roles (108, 112, 115).

Recently, Barth et al. (116) identified an increase in peripheral switched memory B-cells which was associated with reduced odds for disease control rate (DCR) (OR = 0.06, 95% CI = 0.01-0.70, p = 0.025) and an increase in naïve B-cells which was associated with an improved DCR (OR =12.31, 95% CI = 1.13-134.22, p =0.039) in cancer patients on ICI treatments.

Despite the progress made in the understanding of the B cell role in cancer and as predictive biomarkers, studies on the role of B cells as biomarkers of toxicities may be lacking. At the time of this review, one study by Das et al. (87) had reported an association between ICI-induced changes in circulating B cells and high grade irAEs (87). In this study, circulating B cells were analyzed in 39 advanced melanoma patients before and after first cycle of treatment with anti PD-1, anti-CTLA4 or combination therapy. They found a reduction in total circulating B cells (mean fold change, 0.7; p ≤ 0.0001) after one cycle of combination therapy (anti-PD-1/anti-CTLA-4) together with enrichment of plasmablasts (CD19^+^CD27^+^CD38^h^), an increase in plasma CXCL13, and CD21^lo^ PD-1+ B cell subset (enriched in PD-1 expression). CD21^lo^ PD-1+ B cells also expressed CXCR4 and CXCR5 at lower levels compared to CD21^hi^ B cells. Although B cell decline was not recorded in monotherapy (anti-PD-1 and anti-CTLA-4), combination therapy induced changes preceded and correlated with the frequency, timing and maximum grade of toxicity. Combination therapy also resulted in a proliferation of CD4^+^ and CD8^+^ T cells in this study. However, no correlation with risk of irAEs was observed. A similar increase in CD21^lo^ B cells after the first cycle of combination therapy, was reported in 23 renal cell carcinoma patients who experienced irAEs (117). Liver cancer patients treated with anti-PD-1 and tyrosine kinase inhibitors also showed decrease B cells in severe irAE patients (118).

A longitudinal assessment of blood in an advanced lung cancer patient who developed late-onset irAEs on PD-1 inhibition, also reported increases in plasmablasts in circulation (119). Using functional ex vivo assays and mass cytometry analysis, defects in the regulatory B cell repertoire was found to predispose NSCLC patients to the development of immune related toxicity following anti-PD-1(L1) blockade (120). Notably, an attenuated presence of circulatory immunosuppressive B cell subsets (IL-10+, TGF-B+ PDL-1+) was observed in patients that developed toxicities compared to those that did not. Overall, while there is some evidence to support the utility of circulatory B cells as biomarkers of toxicity, more studies need to be conducted to address issues such as the small sample sizes, and to investigate whether B cell changes occur for specific ICI therapy for toxicity prediction. Other studies could also address the dynamics of circulatory and tumor infiltrated B cells as toxicity biomarkers and the mechanistic understanding of these changes.

## Challenges and avenues for application of autoantibodies and B cells as biomarkers of irAEs

One important consideration in any further development of AAbs and B cells as toxicity biomarkers is the need to validate the emergence of potential markers. Currently, many interesting studies have sought to assess differential features among cancer patients with toxicities and those without with encouraging discriminatory benefit. However, this potential has since not been validated for use in the clinical settings. Increased funding for well-developed clinical validation studies will need to be considered to advance this field of research. Also, considering the occurrence of delayed onset irAEs (i.e irAEs that occur > 6-12 months following ICI treatment) (121, 122), there is a limitation of a true control group, to test differential expression of AAb markers in patients (101). Hence, longitudinal studies or landmark analyses will need to be considered in any further studies for a better discrimination of toxicity groups.

High grade irAEs (grade 3-5) present with increased complications as compared to grade 1 and 2 toxicities. Therefore, biomarker discovery studies should be preferably towards markers associated with and of clinical significance for these toxicities. Future studies will need to be well powered or sub-analyzed, with a focus on identifying AAb biomarkers for higher grade irAEs. Currently, it is unlikely that one single AAb marker will be specific or sensitive enough to accurately predict irAEs. Single targets do not possess the sensitivity and specificity to be used effectively as screening biomarkers (123). Fortunately, the emergence of novel proteome microarrays such as the Huprot™ microarray, allow for the detection of a large number of AAb targets at the same time, covering about 81% of the human proteome. By leveraging these technologies, differences in circulating proteins can be identified and validated using multiplex assays or focused arrays for toxicity prediction.

The application of artificial intelligence and robust statistical analysis will also be critical to address the complexity of data generated particularly from proteomics as these techniques have been very useful in the prediction of various diseases (124). AAb markers for anti-PD-1(L1) related irAEs may also be different from anti-CTLA-4 markers. The continuous appreciation of the diverse mechanism of PD-1(L1) and CTLA-4 inhibition in antitumor activity and toxicities, provides enough justification for the biological possibility of this suggestion (125, 126). An impetus for further future direction has been provided by Gowen et al. (94) who reported differential expression of AAb markers of toxicity among patients on anti-PD-1, anti-CTLA-4 and combination therapy.

Many cancer patients that undergo treatments with ICIs may have received prior treatments such as with chemotherapy or radiation therapy which can affect B cells (127, 128). For example, a longitudinal assessment of B cell populations in patients with solid malignancies showed decreases in total and specific B-cell subsets following chemotherapy (128). Hence, studies assessing B cell changes as biomarkers of toxicity will need to consider the possible effects of these prior therapies. While studies on the effect of prior therapies on the risk of irAEs in cancer patients treated with ICIs remain scarce, the sequential treatment of cancer patients with ICIs (3 months following radiotherapy), showed no increased risk for the occurrence of irAEs (129). Whether these therapies can affect B cells and AAb changes for the prediction of ICI-toxicities, will need to be well studied.

It is also possible that organs involved in irAEs may express organ specific AAbs, and therefore sufficiently powered studies in these separate populations should be encouraged. Furthermore, whether AAbs are functionally linked to the development of irAEs or are just bystanders also needs to be determined.

Finally, assessing the kinetics of AAb titers and B cells as a diagnostic adjunct for patients suspected of irAEs, would help clinicians determine whether to continue or hold treatments following irAE onset. B cells and serum AAbs have clinical applications in this view and studies will need to be performed to test this approach. More studies will also be required to identify B cell alterations and to understand the mechanisms of B cell hyperactivity and tolerance imbalance for toxicity prediction.

## Conclusion

In conclusion, given the advances in the research in this field, there is some air of optimism regarding the identification of AAbs and B cells for the prediction and monitoring of irAEs. To achieve this, large clinical studies are required to determine patients who can maximize the benefits of ICIs while minimizing toxicities. There is also the opportunity to assist with clinical surveillance in order to avoid the occurrence of severe, potentially life-threatening side effects. In the future, the focus should be on the development and validation of AAb and B cell biomarkers that are capable of translation into the clinical setting.

## Author contributions

JT, PZ and EG conceived the idea of this review. JT wrote the draft. PZ, EG and AG reviewed the manuscript and provided insights. All authors contributed to the article and approved the submitted version.
